# Tribological Performance of FFF-Printed PLA and PLA-Wood Parts: The Effects of Infill Architecture, Heat Treatment, and Water Absorption

**DOI:** 10.3390/polym18192324

**Published:** 2026-09-23

**Authors:** Berkay Ergene, Bayram Yıldız

**Affiliations:** 1 Department of Mechanical Engineering, Faculty of Technology, Pamukkale University, Denizli 20160, Türkiye; 2 Department of Metallurgical and Materials Engineering, Faculty of Technology, Pamukkale University, Denizli 20160, Türkiye; bayramyildiz@pau.edu.tr

**Keywords:** polylactic acid, polymer composite, additive manufacturing, coefficient of friction, wear resistance, annealing, water exposure, post-process

## Abstract

Polymer components fabricated via fused filament fabrication (FFF) are frequently exposed to friction and wear under diverse environmental conditions in various engineering applications. The tribological performance of these components is influenced by both the printing parameters employed during fabrication and subsequent post-processing and service conditions. Previous studies have generally investigated the effects of printing parameters or post-processing steps separately. However, a comprehensive evaluation of in-service performance requires the sequential consideration of these factors. This study aimed to evaluate the tribological performance of FFF-printed PLA and PLA-Wood specimens through a sequential approach considering the infill architecture, heat treatment, and subsequent water absorption. The findings indicate that the best-performing infill architecture among the investigated configurations was the rectilinear pattern at an 80% infill density, which exhibited the lowest volumetric wear loss among all printing configurations. The best-performing heat treatment conditions varied by material and were determined to be 90 °C for 15 min for PLA and 90 °C for 45 min for PLA-Wood, reducing volumetric wear loss from 17.97 to 14.32 mm^3^ (approximately 20%) and 24.92 to 22.36 mm^3^ (approximately 10%), respectively. Moreover, water absorption largely eliminated the heat-treatment-induced improvement in PLA, with volumetric wear loss increasing to 17.93 mm^3^, whereas the improvement was substantially retained in PLA-Wood, which exhibited a wear loss of 22.67 mm^3^ after water absorption. Overall, the findings demonstrate that improvements in wear resistance achieved through processing may not be equally retained after subsequent environmental exposure and that this response depends on the material composition.

## 1. Introduction

Throughout history, technological advancements have continually evolved in line with human needs and expectations. Emerging technologies have profoundly influenced not only manufacturing methods but also the fields of materials science and engineering. Polymers, which hold a significant place among today’s engineering materials, have become fundamental components of numerous industrial and everyday products owing to their advantages, including a low density, chemical resistance, high formability, and cost-effective production [1,2]. The performance of polymers in engineering applications largely depends on achieving the desired mechanical, thermal, and tribological properties. To this end, polymer materials are sometimes optimized through topological design and, in other cases, produced as composites by incorporating various reinforcing agents or fillers [3]. The incorporation of carbon fibers, glass fibers, metal powders, or wood particles can significantly alter the properties of the matrix material, including the strength, stiffness, thermal conductivity, and wear resistance [4,5,6].

In addition to these developments, additive manufacturing (AM) technologies have rapidly gained prominence as a means of overcoming the limitations of traditional manufacturing methods, particularly for producing parts with complex geometries. AM enables the direct fabrication of three-dimensional objects from computer-aided models through layer-by-layer deposition [7]. This approach offers significant advantages for rapid prototyping, lightweight structural components, and customized designs. Among AM techniques, fused filament fabrication (FFF) has become one of the most widely used methods due to its low equipment cost, accessibility, and compatibility with a broad range of thermoplastic materials [8,9]. Beyond conventional thermoplastics such as polyethylene terephthalate glycol (PETG), acrylonitrile butadiene styrene (ABS), acrylonitrile styrene acrylate (ASA), polyamide (PA), polycarbonate (PC), and polylactic acid (PLA), FFF also enables the fabrication of composite variants reinforced with carbon fibers, glass fibers, wood powder, metal particles, or mineral fillers [10,11,12,13]. Among these materials, PLA is preferred in general prototyping and educational applications due to its biodegradable nature, dimensional stability, and low printing temperature, whereas PLA-Wood finds widespread use in fields such as architectural modeling, decorative product design, and industrial design thanks to its aesthetic surface texture and low density [14,15]. Beyond these primarily aesthetic applications, PLA-Wood has also been investigated for functional components such as furniture connectors [16], suggesting its potential for low-load components involving repeated contact or relative motion. In such applications, joints, connectors, and other contact elements may experience friction and progressive surface damage during use. Moreover, using additively manufactured polymer components in marine and other moisture-exposed environments [17] adds another consideration, since contact and wear may occur in the presence of environmental moisture.

Studies on the mechanical and tribological behavior of polymer and polymer-composite parts produced using the FFF technique demonstrate the decisive influence of manufacturing parameters on the parts’ final properties. The literature reports that parameters such as the printing temperature, layer thickness, infill density, printing speed, and build orientation significantly affect the part density, surface roughness, coefficient of friction (COF), and wear resistance [18,19,20,21]. In light of these findings, numerous studies have further examined how specific FFF parameters govern friction and wear behavior across varying material systems and test conditions. Bolat et al. [22] attempted to predict the wear and friction behavior of recycled-ABS parts produced via FFF with varying layer thicknesses, infill densities, and build orientations using an artificial neural network approach. In another study [23], the effects of the layer thickness, infill density, and build direction on the dry sliding wear performance of PETG parts were examined. The authors reported a positive interaction between volume loss and layer thickness, while no direct interaction was observed between the infill density/build direction and the COF. Building upon the broader understanding of parameter-dependent wear behavior, Yaman et al. [24] evaluated the influence of the build orientation on PLA-matrix parts containing copper, brass, bronze, tungsten, and carbon fiber and identified that tungsten-containing PLA parts printed in the flat orientation exhibited the highest wear resistance. In related work, Rashia et al. [25] showed that glass-fiber-added PLA samples produced with a raster angle of 0° had the lowest wear. Additionally, Maguluri et al. [18] used a Taguchi design to quantify the influence of individual FFF parameters on the wear behavior of PLA biopolymer parts and to identify parameter combinations that minimize wear. Furthermore, Raichur et al. [26] demonstrated that adding 5% and 10% nano-clay to PLA significantly reduced wear, while Abdulla et al. [27] reported that higher sliding velocities, higher infill densities, and greater test forces decreased the specific wear rate of 3D-printed PLA samples.

Several researchers have aimed to enhance the performance of FFF-printed parts by applying post-processing treatments to improve material properties [28,29]. Ergene et al. [28] investigated the water absorption behavior of PLA and carbon-fiber-reinforced PLA parts printed with different infill densities by exposing them to seawater for various durations and examined how water exposure influenced their subsequent wear performance. Building on the growing interest in post-processing-based performance improvement, Bolat and Ergene [29] analyzed the effects of the test force and post-printing heat treatment on the wear behavior of neat, glass-fiber-added, and carbon-fiber-added PA6 parts produced via FFF. Likewise, Karthick et al. [30] applied annealing and laser treatment to glass-fiber-reinforced PA6 produced through FFF and evaluated the wear performance under different test forces and sliding velocities, reporting that laser-treated samples exhibited the highest wear resistance and lowest friction due to densified interlayer fusion and surface texturing, while annealed specimens showed moderate improvements. In a related study, Soundararajan et al. [31] examined the influences of heat treatment at 70, 140, and 210 °C on the wear behavior of carbon-fiber-reinforced PA6 parts, finding that treatments at 70 and 140 °C enhanced wear resistance owing to improved interlayer bonding, whereas no such improvement occurred at 210 °C. Beyond annealing, alternative thermal post-processing routes have also been explored. Gul et al. [32] demonstrated that salt remelting treatment applied to FFF-printed polyethylene terephthalate (PET) at 170–220 °C significantly altered the wear behavior, with the treatment at 210 °C reducing the wear rate by 74.2%. Finally, Prabakaran et al. [33] studied various laser surface-polishing parameters applied to 3D-printed ASA parts and reported a 64.2% reduction in the wear rate for laser-polished specimens.

Although numerous studies have investigated the tribological performance of FFF-printed polymers, most prior research has focused on either the printing parameters or post-processing treatments individually. Hence, the combined and sequential effects of the printing architecture and subsequent post-processing treatments on the tribological behavior of wood-filled PLA (PLA-Wood) remain insufficiently explored. To address this research gap, the current study systematically evaluates PLA and PLA-Wood specimens by first analyzing the effects of the infill density and pattern and then examining heat treatment and water absorption within a unified experimental framework. In contrast to previous studies, this work assesses the influence of manufacturing parameters and post-processing treatments consecutively using the same material system, thereby enabling a comprehensive evaluation of their individual and cumulative contributions to tribological performance. This sequential approach further enables an evaluation of whether the tribological improvement achieved through heat treatment is retained after subsequent environmental exposure and whether this response differs between PLA and PLA-Wood. By integrating COF measurements, volumetric wear analysis, and worn-surface characterization, this work aims to identify processing conditions that improve the tribological performance of PLA and PLA-Wood parts and to deepen the understanding of the mechanisms governing their friction and wear behavior.

## 2. Materials and Methods

### 2.1. Materials

Neat PLA filament and PLA-Wood filament containing 30% wood filler were used as the raw materials in the present study. Both filaments were commercially sourced from Porima (Çiftlikköy/Yalova, Türkiye) and had a nominal diameter of 1.75 mm. The PLA-Wood filament was used as received, and no additional characterization of wood-filler dispersion within the PLA matrix was performed in this study. To reduce the moisture content and prevent related defects during FFF processing, PLA and PLA-Wood filaments were dried in a drying oven (Miprolab MLF-120, Mipro Laboratuvar Cihazları Üretim A.Ş., Yenimahalle, Ankara, Türkiye) at 40 °C or 4 h before printing. Table 1 summarizes the physical and mechanical properties and recommended processing parameters for the PLA and PLA-Wood filaments, as provided by the manufacturer.

### 2.2. Experimental Workflow

The experimental workflow of this research is illustrated in Figure 1. The experimental design consists of three main stages:

- Identifying the best-performing infill architecture among those investigated based on the samples’ tribological response.
- Identifying the best-performing heat treatment conditions among those investigated based on the tribological response of the selected specimen identified in the first stage.
- Evaluating the effect of water absorption on the tribological performance of both untreated and heat-treated selected specimens.

In the first stage, wear specimens were produced using PLA and PLA-Wood filaments with different infill densities and infill patterns. The COF and volumetric wear loss of the produced specimens were determined, and based on these results, the infill architecture that provided the best tribological performance was selected for the subsequent heat-treatment stage. Various experimental design approaches and Taguchi-based analyses have been employed in the literature to identify the optimal processing conditions or best-performing parameter combinations [34,35]; in the present study, the best-performing configuration was selected based on the lowest volumetric wear loss, with the corresponding COF results used as supporting tribological evidence. In the second stage, specimens with the selected infill architecture were reprinted and subjected to heat treatment at different temperatures for varying durations. Tribological tests conducted after heat treatment were used to identify the best-performing temperature–duration combination among those investigated. In the third stage, untreated specimens with the selected infill architecture and specimens that had undergone the selected heat treatment were soaked in water. Following water absorption, the tribological behavior of the specimens was re-evaluated, and the wear mechanisms were examined for all samples studied. Thus, the individual and sequential effects of production parameters, heat treatment, and water absorption on the tribological performance of PLA and PLA-Wood parts were systematically assessed.

### 2.3. Sample Design and 3D Printing Process

Wear test specimens with a diameter of 10 mm and a height of 20 mm, conforming to ASTM G99, were designed in SolidWorks 2022 (Figure 2a). The design was then exported in “.stl” format and transferred to the slicing software (ElegooSlicer 1.1.8.2 version), where it was positioned on the virtual build platform (Figure 2b). The generated G-code files were subsequently transferred to a 3D printer. All samples were produced using a commercially available 3D printer with a closed frame (Elegoo Centauri Carbon, Elegoo, China) to maintain a stable thermal condition during printing (Figure 2c). All printing parameters were held constant across samples to ensure experimental consistency, with only the infill density (20%, 50%, and 80%) and infill pattern (gyroid, honeycomb, and rectilinear) varied. The investigated infill densities and patterns were selected following a detailed review of previous FFF studies to provide representative low, intermediate, and high infill levels and to compare commonly used architectures with different filament-path arrangements. No formal design-of-experiments approach or preliminary optimization study was used for parameter selection. The remaining printing parameters were kept constant based on the recommended processing conditions of the commercial filaments and commonly reported FFF processing conditions in the literature. The complete set of printing parameters used to produce the wear test samples (five specimens per experimental set) is summarized in Table 2. Upon the completion of printing, samples were allowed to cool naturally in the build chamber before being removed from the build platform. Finally, all printed specimens were visually inspected, and no printing defects such as warping, delamination, or incomplete extrusion were observed. Representative FFF-printed PLA and PLA-Wood wear test specimens fabricated with different infill architectures are shown in Figure 2d.

### 2.4. Heat Treatment Procedure

In the first stage, PLA and PLA-Wood samples produced with different infill patterns (gyroid, honeycomb, and rectilinear) and infill densities (20%, 50%, and 80%) were evaluated tribologically. Based on the resulting volumetric wear loss data, all nine infill architecture combinations were compared, and the 80% rectilinear configuration, which exhibited the lowest volumetric wear loss for both materials, was selected for the subsequent heat-treatment stage. Thus, the rectilinear pattern was not selected a priori but was identified from the first-stage experimental results. Samples reprinted using this selected infill architecture were heat-treated at 70, 80, and 90 °C for 15, 30, and 45 min. Previous studies indicate that annealing is typically conducted above the glass transition temperature, where molecular rearrangement and crystallization may occur without melting the polymer [36,37]. Accordingly, heat-treatment temperatures of 70, 80, and 90 °C were selected to provide thermal exposure above the glass-transition temperatures of both materials while remaining well below the melting region. Treatment durations of 15, 30, and 45 min were investigated to evaluate the influence of different thermal exposure times within this temperature range. The heat treatment was performed in a drying oven (Miprolab MLF-120). After heat treatment, the samples were allowed to cool naturally to room temperature and were then subjected to tribological tests. The obtained tribological results were compared, and the temperature–duration combination that produced the lowest volumetric wear loss for each material was selected for the subsequent water-absorption stage. Thus, the selected heat-treatment conditions were determined from the tribological results obtained from the nine investigated temperature–duration combinations rather than being predetermined.

Accordingly, heat-treatment temperatures of 70, 80, and 90 °C were selected to provide thermal exposure above the glass-transition temperatures of both materials while remaining well below the melting region. Treatment durations of 15, 30, and 45 min were investigated to evaluate the influence of different thermal exposure times within this temperature range.

### 2.5. Water Absorption Procedure

After selecting the best-performing heat treatment condition for each material in the second stage, the third stage assessed the impact of water absorption on tribological performance. Untreated samples fabricated with the selected infill architecture, as well as samples subjected to the selected heat treatment, were immersed in distilled water for 15 days. The specimens remained fully submerged in distilled water throughout the entire 15-day conditioning period. After immersion, specimens were removed and allowed to equilibrate at ambient conditions for 10 min, and the surface water was removed with a paper towel. The water absorption (%) of the specimens was calculated using Equation (1) based on the measured masses before and after water immersion. All water-absorbed samples were subsequently subjected to tribological testing to evaluate the influence of water absorption on the tribological behavior of both heat-treated and untreated samples.

(1) $$Water\;Absorption\;(\%)=\frac{M_{f}-M_{i}}{M_{i}}\times 100$$

where *M_f_* and *M_i_* represent the final and initial masses of the FFF-printed parts, respectively.

### 2.6. Characterization

The tribological performance of the samples produced was investigated at room temperature (23 ± 2 °C) and a relative humidity of approximately 40% under dry sliding conditions using a pin-on-disc tribometer (TURKYUS PODWT, TurkYus Makina Ar-Ge Üretim Teknolojileri, Nilüfer, Bursa, Türkiye), as shown in Figure 3.

A 600-mesh silicon carbide (SiC) abrasive paper, with a measured surface roughness (Ra) of 5.49 ± 0.36 µm, determined from ten measurements at different surface locations, was used as the counterface to provide controlled abrasive contact. This intermediate abrasive grade was selected to enable measurable material removal while avoiding the severe abrasion associated with substantially coarser abrasive surfaces. Tests were performed under a constant normal load of 10 N at a sliding speed of 2 m/s for 90 s, corresponding to a total sliding distance of 180 m. The friction force was continuously recorded throughout the test (Esit Data Logger 1.1.8 software), and the COF was calculated from the collected data. The masses of the specimens were measured before and after each wear test using a precision balance (Radwag AS/220/C/2). Following the wear test, the specimens were cleaned to remove wear debris, and final mass measurements were then taken. Mass losses were divided by specimen densities to determine the volumetric wear loss. Equations (2) and (3) were used, respectively, to calculate the COF and volumetric wear loss. For each experimental condition, five independently printed specimens were tested. The reported COF and volumetric wear loss values are presented as the mean ± standard deviation (n = 5), and the error bars in the corresponding figures represent the standard deviation.

(2) $$Coefficient\;of\;Friction=\frac{Frictional\;Force\;(N)}{Normal\;Force\;(N)}$$

(3) $$Volume\;Loss\;({mm}^{3})=\frac{Mass\;Losss\;(g)}{Density\;(g/{mm}^{3})}$$

The worn surfaces of the specimens were examined using a field-emission scanning electron microscope (FESEM, Gemini, Carl Zeiss, Germany).

## 3. Results and Discussion

### 3.1. Effect of Infill Density and Pattern on Tribological Performance

The experimental workflow (Figure 1) outlines the initial stage of this research, which aimed to identify the best-performing infill architecture for PLA and PLA-Wood specimens. To this end, specimens were fabricated using three infill densities (20%, 50%, and 80%) and three infill patterns (gyroid, honeycomb, and rectilinear). Their tribological response was then evaluated. The COF results of FFF-printed PLA and PLA-Wood samples are shown in Figure 4. It can be pointed out that both production parameters affect the COF values. For both PLA and PLA-Wood samples, the COF increases with the infill density, regardless of the infill pattern. In PLA samples produced with different patterns, the COF value, which ranged from 0.431 ± 0.015 to 0.493 ± 0.021 when the infill density was 20%, increased to 0.502 ± 0.022 − 0.557 ± 0.017 as the infill density rose to 80% (Figure 4a). Similarly, in PLA-Wood specimens, increasing the infill density from 20% to 80% led to a rise in COF from 0.671 ± 0.024 − 0.722 ± 0.029 to 0.742 ± 0.017 − 0.822 ± 0.033 (Figure 4b). The observed increase in the COF with a higher infill density can be attributed to changes in the internal structural continuity of the FFF-printed specimens. As the infill density increases, the void volume within the structure decreases, resulting in a more continuous filament network beneath the contact surface [38,39]. This improved continuity enhances structural support during sliding and diminishes local deformation under the applied normal load. Therefore, the effective load-bearing contact area between the specimen and the abrasive counterface may increase, which leads to greater resistance to sliding and, thus, a higher COF. The COF of FFF-printed components is significantly affected by tribological test parameters, such as the applied normal load, sliding speed, sliding distance, counterface material, and lubrication environment [29,40,41]. As a result, the literature reports a broad range of COF values for FFF-printed PLA and PLA composite samples, typically between 0.2 and 0.8 under various experimental conditions [28,42,43,44]. In this study, COF consistently increased with an increasing infill density for both PLA and PLA-Wood samples, regardless of the infill pattern. Yet, previous studies have documented different trends between the infill density and COF; some studies report a decrease in COF with an increasing infill density [45,46], while others find no significant correlation [28,47].

Although the effect of the infill pattern on the COF was less pronounced than that of the infill density, it remained noticeable for both materials. In PLA, the lowest COF was recorded with the rectilinear pattern (0.431 ± 0.015 at a 20% infill density), followed by the honeycomb (0.474 ± 0.020 at a 20% infill density) and gyroid patterns (0.493 ± 0.021 at a 20% infill density), respectively. Similarly, the highest COF in PLA-Wood samples was observed with the gyroid pattern (0.722 ± 0.029 at a 20% infill density). Although the rectilinear and honeycomb patterns yielded comparable results in PLA-Wood samples, the honeycomb pattern exhibited a slightly lower COF. The COF values for rectilinear and honeycomb patterns obtained at a 20% infill density were 0.686 ± 0.021 and 0.671 ± 0.024, respectively. While the samples were printed at the same infill density, different pattern arrangements can lead to distinct load-transfer paths and stiffness distributions [48]. Compared with other patterns, the gyroid structure offers a continuous, highly interconnected geometry. Therefore, despite having the same fill density, the gyroid pattern may provide a larger effective contact area and more stable load transfer during sliding. This may explain why the gyroid structure consistently exhibits higher COF values.

A comparison of the two materials revealed that PLA-Wood samples exhibited higher COF than PLA across all printing conditions. This can be attributed to the wood particles in the polymer matrix, which may alter the surface topography and roughness of 3D-printed samples [49], thereby increasing interactions with the abrasive surface. As illustrated in Figure 2d, to emphasize the effects of the infill density and pattern, the surfaces of samples designated for wear testing were intentionally left uncovered, without a top layer. Hence, surface roughness measurements were not feasible because the surfaces were unsuitable for this analysis. However, previous studies showed that adding wood powder increases the surface roughness of printed samples [50], which may enhance interfacial friction during sliding. Furthermore, variations in hardness may contribute to this observed difference [51]. The incorporation of wood particles locally increases the hardness of the contact surface and modifies the interaction, potentially resulting in a distinct friction response compared to PLA.

To further illustrate the evolution of friction during the wear test, Figure 5 presents representative COF–time curves for PLA and PLA-Wood specimens with the 80% rectilinear infill architecture. Both materials exhibited an initial transient period, characterized by a rapid change in COF during the early stage of sliding. This was followed by a transition stage, after which the COF fluctuated around a relatively stable level for the remainder of the test. The curves therefore indicate that a relatively stable frictional regime was established for both PLA and PLA-Wood during the 90 s sliding period.

Figure 6 shows the volume loss of 3D-printed PLA and PLA-Wood samples fabricated with different infill densities and patterns. Regardless of the material, both parameters had a remarkable effect on wear performance. Increasing the infill density reduced volume loss across all cases, indicating improved tribological performance. For PLA samples, as the infill density increased from 20% to 80%, the volume loss of specimens with a gyroid pattern decreased from 51.09 ± 2.14 mm^3^ to 22.27 ± 0.88 mm^3^ (Figure 6a). Similar decreases were observed for the honeycomb and rectilinear patterns, with the volume loss dropping from 42.36 ± 5.40 mm^3^ to 19.31 ± 1.71 mm^3^ and from 55.66 ± 1.35 mm^3^ to 17.97 ± 0.92 mm^3^, respectively. Although increasing the infill density reduced volume loss, it should be noted that the distinctions between infill patterns became less noticeable at the highest density. PLA-Wood samples also showed a similar trend (Figure 6b). The volume loss decreased from 78.23 ± 0.40 mm^3^ to 28.42 ± 1.06 mm^3^ for the gyroid pattern, from 63.36 ± 0.40 mm^3^ to 25.46 ± 1.60 mm^3^ for the honeycomb pattern, and from 85.87 ± 9.51 mm^3^ to 24.92 ± 0.63 mm^3^ for the rectilinear pattern as the infill density increased from 20% to 80%. The improvement in wear resistance due to an increased infill density can be attributed to the formation of a more continuous load-bearing structure within the printed specimen. Increasing the infill density reduces the internal void content and results in a more structurally continuous filament network, which enables the applied load to be transferred through a greater number of interconnected filament paths. This uniform distribution of the load throughout the structure prevents concentration in localized regions [52]. Enhanced structural continuity also provides improved subsurface support beneath the worn surface, reducing susceptibility to localized penetration by abrasive SiC asperities during sliding. Furthermore, reducing internal voids minimizes potential stress-concentration sites, thereby limiting local damage accumulation and reducing the possibility of material removal under abrasive conditions. Conversely, structures with lower infill densities contain larger gaps between adjacent filament pathways, thereby restricting efficient load transfer and promoting localized deformation under contact loading [52,53]. The trend observed in this study is consistent with previous tribological investigations of FFF-printed polymers. For instance, Ergene et al. demonstrated that regardless of environmental exposure, increasing the infill density from 20% to 100% consistently reduced volume loss in both PLA and carbon fiber-reinforced PLA [28]. Similarly, Sagar et al. recorded a 93% decrease in the specific wear rate of PLA samples as the infill density transitioned from a hollow (0%) to a solid (100%) configuration, which they attributed to improved material continuity beneath the worn surface, reduced void-induced stress concentrations, and enhanced thermal conductivity resulting from decreased porosity [46]. This trend was further supported by Şirin et al., whose findings indicated that mass loss was minimal in the specimens printed with a 70% infill density, the highest density tested [45]. Their results also revealed a clear correlation between decreasing the material loss and increasing the Shore D hardness with an increasing infill density. The wear results of the current study align with previous observations and further confirm that increasing the infill density provides benefits for both neat PLA and PLA-Wood.

When the effect of the infill pattern on wear behavior is examined, the results indicate that it depends on the infill density. Apparent discrepancies in wear performance were observed among infill patterns at low infill densities, but these differences were diminished considerably as infill density increased. Moreover, while the honeycomb pattern exhibited the lowest volume loss at low infill densities, the rectilinear pattern yielded the highest wear resistance at the highest infill density. For instance, at an infill density of 20%, the PLA specimen with a honeycomb pattern showed a volume loss of 42.36 ± 5.40 mm^3^, while the corresponding rectilinear specimen exhibited a volume loss of 55.66 ± 1.35 mm^3^ (Figure 6a). The volume loss of PLA-Wood samples with honeycomb and rectilinear patterns was recorded as 63.39 ± 0.40 mm^3^ and 85.87 ± 9.51 mm^3^, respectively (Figure 6b). Even when all specimens are produced with the same infill density, different infill patterns during fabrication result in distinct internal structures. The shape and distribution of voids, the number of raster nodes, the load-transfer path, and the local stiffness and stress distributions all vary with the infill pattern. Hence, these structural differences likely account for the observed variations in volumetric wear loss among the infill patterns.

The comparison of the wear performance of PLA and PLA-Wood samples proves that PLA-Wood samples exhibited higher volumetric wear loss across all printing conditions. The most pronounced difference occurred at low infill densities. For example, the volumetric loss difference between PLA and PLA-Wood samples with the rectilinear pattern was 30.21 mm^3^ at a 20% infill density and decreased to 6.95 mm^3^ at a 80% infill density. This trend was consistent across other infill patterns. For the gyroid pattern, the difference between PLA and PLA-Wood decreased from 27.13 to 6.15 mm^3^. In the honeycomb pattern, the difference decreased from 21.0 to 6.15 mm^3^.

Volumetric wear loss results showed that an 80% infill density and a rectilinear pattern provided the highest wear resistance, regardless of the material. These parameters were therefore selected for subsequent heat-treatment and water absorption studies.

### 3.2. Effect of Heat Treatment on Tribological Performance

Heat treatment is widely used to improve the mechanical and tribological performance of polymer materials [54]. In this study, after identifying the best-performing infill architecture among those investigated, heat treatment was applied to specimens with the selected infill architecture at different temperatures and durations. The effect of heat treatment on the tribological behavior of these materials was then examined.

Figure 7 presents the COF values of PLA and PLA-Wood specimens following heat treatment. Heat treatment had a remarkable effect on the COF, with both temperature and duration playing critical roles, as did the material type. For untreated PLA, the COF value was 0.502 ± 0.022, and only minor changes were observed at lower temperatures and shorter processing times, with values remaining largely unchanged. However, increasing the temperature to 90 °C and subjecting the samples to 30 and 45 min heat treatments resulted in approximately 15 and 62% higher COF values, respectively. In contrast, the PLA-Wood sample exhibited a different trend. Although the COF generally increased following heat treatment at 70 and 80 °C, lower COF values were obtained after heat treatment at 90 °C for 15 and 45 min. The lowest COF value, 0.678 ± 0.008, was obtained after a 45 min heat treatment at 90 °C. As detailed later, the volumetric wear loss results also indicate that the 45 min treatment at 90 °C produced the lowest volumetric wear loss among the investigated heat-treatment conditions. This alignment suggests that a more stable tribological surface is established under these heat treatment conditions. Potential molecular rearrangement and improved matrix continuity, resulting from thermal effects [54], may have contributed to reduced deformation and material loss. Therefore, the simultaneous decrease in COF and wear loss indicates that the 45 min treatment at 90 °C offers the most favorable tribological response for PLA-Wood under the investigated conditions. For PLA, since the COF remains nearly unchanged at lower temperatures, short-term treatments at 70 °C may be preferable for energy and time efficiency.

Figure 8 shows the volumetric wear loss in 3D-printed PLA and PLA-Wood samples after heat treatment. The results indicate that the heat treatment process produces varying outcomes depending on the material type, with the effect governed by the interaction between the duration and temperature. An analysis of the wear performance of 3D-printed PLA specimens (Figure 8a) demonstrates that heat treatment generally reduces wear-induced material loss and enhances wear resistance. Specifically, 15 and 30 min heat treatments positively influence the wear behavior, and increasing the applied temperature from 70 °C to 90 °C further reduces wear. The lowest wear rate, 14.32 ± 0.26 mm^3^, was achieved with a 15 min heat treatment at 90 °C, corresponding to a reduction of nearly 20% compared to the untreated sample. Similarly, heat treatments at 80 °C for 30 min (14.82 ± 0.56 mm^3^) and at 90 °C for 30 min (14.79 ± 1.19 mm^3^) yielded significant improvements, with reductions of approximately 17.5%. In contrast, extending the heat treatment duration to 45 min generally reversed the decreasing trend in wear volume. For example, heat treatment at 80 °C for 45 min increased the wear volume to 18.65 ± 0.67 mm^3^ compared with the untreated sample. Therefore, it can be deduced that prolonged heat-treatment durations can negatively affect the material’s wear behavior, indicating that the wear response depends on the specific temperature–duration combination. The observed enhancement in wear resistance may be explained by the microstructural changes expected during heat treatment. Previous studies show that heat treatments conducted above the glass transition temperature on FFF-fabricated PLA specimens can promote molecular rearrangement [55], reduce residual stresses generated during printing, and increase the degree of crystallization [56]. These literature-reported changes may improve the structural integrity of the deposited filament network and promote more effective load transfer, which may contribute to the lower material removal observed under some heat-treatment conditions. Moreover, the findings reveal that extended thermal exposure does not consistently improve tribological performance. Prolonged thermal exposure may cause structural alterations that could partially eliminate or reduce the effectiveness of the beneficial microstructural changes provided by heat treatment [54]. Under such conditions, an increase in the wear rate may be observed with certain temperature–duration combinations. Accordingly, the results indicate that the PLA wear response depends on the specific temperature–duration combination and that longer thermal exposure does not necessarily provide greater wear resistance.

An analysis of the heat-treatment process for the wear behavior of PLA-Wood specimens (Figure 8b) shows a markedly different response from that of PLA. The volumetric wear loss of the untreated material was 24.92 ± 0.63 mm^3^, and this value was exceeded under nearly all heat-treatment conditions. Improved wear performance was observed only when samples were heat-treated at 70 °C for 15 min or at 90 °C for 45 min. The lowest wear volume, 22.36 ± 0.58 mm^3^, was recorded at 90 °C for 45 min, indicating an improvement of nearly 10%. In contrast, a 30 min heat treatment at 90 °C resulted in the highest wear volume among all combinations (37.01 ± 1.96 mm^3^), indicating significant deterioration in wear performance. Wear performance exhibited a strong dependence on the time–temperature interaction, with a 30 min heat treatment at any temperature significantly reducing it. Moreover, no clear or consistent relationship was identified between the heat treatment temperature and wear behavior.

The results indicate that the tribological response cannot be attributed solely to the duration or temperature of heat treatment. Instead, wear behavior is determined by the interaction between these two parameters and may also vary with the material composition. For PLA, a consistent improvement in wear behavior is observed across most heat-treatment conditions, suggesting a broad and practical processing window. In contrast, PLA-Wood is highly sensitive to heat treatment and requires a narrower processing window. Therefore, selecting heat-treatment conditions should consider the material-specific tribological response. According to post-heat-treatment wear data, 90 °C for 15 min and 90 °C for 45 min produced the most favorable results in this study for PLA and PLA-Wood, respectively, and these specimens were selected prior to the water-absorption process.

### 3.3. Effect of Water Absorption on Tribological Performance

Water absorption differed considerably between PLA and PLA-Wood. After 15 days of water immersion, water absorption was 0.6% for untreated PLA and 1.73% for PLA-Wood. For the heat-treated specimens, the corresponding values were 0.4% for PLA and 1.55% for PLA-Wood. Thus, PLA-Wood exhibited substantially higher water absorption than PLA regardless of the prior heat-treatment condition.

The effect of water absorption on the tribological performance of the 3D-printed PLA and PLA-Wood samples is presented in Figure 9. The influence of water absorption on friction behavior varied by material (Figure 9a). Minimal changes in COF were observed in PLA samples after water absorption, whereas PLA-Wood samples exhibited a significant decrease. The COF of untreated PLA was 0.502 ± 0.022 and remained nearly unchanged at 0.508 ± 0.009 after water exposure. Heat-treated PLA exhibited a COF of 0.523 ± 0.003 after water absorption. In contrast, the COF of PLA-Wood exposed to water decreased by nearly 15% compared to the untreated sample (0.634 ± 0.003). The COF of heat-treated PLA-Wood decreased from 0.678 ± 0.008 to 0.635 ± 0.027, also representing a reduction of approximately 7%. This difference in the friction response between PLA and PLA-Wood can be attributed to water molecules retained by the wood particles and the wood particle–polymer matrix interfaces, as well as within the PLA matrix. Hence, the greater moisture absorption of PLA-Wood may have altered the mechanical behavior of the near-surface region to a greater extent. The excess water may have increased plasticization in the near-surface region and reduced local stiffness. Therefore, the resistance between the material and the counterface decreased, resulting in a significant drop in COF. Since the PLA specimens absorbed less moisture, there was no significant change in the mechanical properties in the near-surface region, and COF remained close to its initial value. Therefore, the contribution of wood fillers to moisture absorption can be considered one of the key factors determining the material’s tribological behavior [28,57,58]. In addition, the nearly identical COF values obtained in both heat-treated and untreated PLA-Wood samples after water absorption suggest that water absorption has a stronger effect on friction behavior than the heat treatment applied prior to water absorption.

Figure 9b compares the impact of water absorption on volumetric wear loss in both untreated and heat-treated materials. As discussed earlier, the selected heat-treatment procedure varied for each material, resulting in different outcomes. After the selected heat treatment, volumetric wear loss decreased by approximately 20% for PLA and 10% for PLA-Wood (Figure 9b). The impact of water absorption also differed by material. After 15 days of water immersion, wear loss in untreated PLA samples changed from 17.97 ± 0.92 to 18.60 ± 0.18 mm^3^. Similarly, the volumetric wear loss of PLA-Wood remained comparable after water absorption, changing from 24.92 ± 0.63 to 24.37 ± 1.21 mm^3^. The most notable finding was observed in samples subjected to both heat treatment and water absorption. The tribological benefit of heat treatment was lost in PLA samples after water absorption, whereas it was largely retained in PLA-Wood samples. The almost 20% improvement in PLA wear resistance due to heat treatment was largely negated by water absorption, with volumetric wear loss returning to levels similar to those of untreated samples. Conversely, the volumetric wear loss of heat-treated PLA-Wood remained nearly unchanged after water absorption at 22.67 ± 0.80 mm^3^, remaining lower than that of untreated samples. This behavior can be attributed to the distinct interactions between water molecules and the two materials. Considering the substantially higher water absorption of PLA-Wood reported above, a greater performance loss would be expected if the observed wear response was governed primarily by the amount of absorbed water. However, the results indicate that tribological performance is influenced not only by the quantity of water absorbed but also by its distribution within the material. Water molecules may have induced plasticization by increasing chain mobility in the PLA matrix [58]. As a result, the load-bearing capacity of the crystalline structure and the enhanced interlayer bonding achieved through heat treatment were reduced, thereby negating the positive effect of heat treatment on wear behavior. In PLA-Wood samples, the higher water content led to water retention within the PLA matrix and wood particles, as well as at the particle–matrix interfaces. Therefore, the plasticization effect in the polymer matrix likely remained limited despite the elevated water content. In summary, while water absorption limits the tribological improvements afforded by heat treatment in both materials, the extent of this effect depends on the material’s internal structure and the distribution of water within it.

### 3.4. Worn-Surface Morphology and Wear Mechanisms

Representative SEM micrographs of the worn surfaces are given in Figure 10. Directionally aligned grooves were observed in all experimental groups, indicating that abrasive wear, particularly micro-scratching and micro-ploughing, was an important component of the material-removal process. Localized cavities, flake-like detachments, accumulated debris, and opened inter-road regions further demonstrate that abrasion was accompanied by localized pull-out and delamination. Directional grooves, microfractures, and wear debris are commonly reported as characteristic morphological indicators of the mixed-wear response in FFF-manufactured PLA-based materials. Moreover, voids between deposited roads can act as preferential damage sites and may retain detached particles, thereby promoting secondary or third-body abrasion.

The untreated PLA surface in Figure 10A was characterized by relatively uniform and fine abrasive grooves. Localized cavities and accumulated debris were primarily concentrated along distinct linear boundaries, which are considered to correspond to inter-road regions or pre-existing FFF voids exposed during sliding. The limited size of the detached regions and the otherwise continuous surface morphology indicate a mild-to-moderate abrasive wear regime, accompanied by localized inter-road opening and material pull-out. This morphology is consistent with the intermediate volume loss measured for untreated PLA.

In comparison, untreated PLA-Wood exhibited substantially more heterogeneous surface damage, as shown in Figure 10B. A large irregular cavity, partially lifted surface regions, and considerable debris accumulation were observed. The morphology indicates that material removal proceeded through a combination of abrasive grooving, localized delamination, and the pull-out of particle-rich regions. The fragments retained within the cavity could subsequently participate in third-body abrasion. The more extensive and irregular material detachment provides a morphological explanation for the considerably higher volume loss of untreated PLA-Wood relative to untreated PLA.

Water-absorbed PLA exhibited dense parallel grooves, inter-road separation, and small flake-like detachments (Figure 10C). Compared with untreated PLA, the damage appeared to be more strongly localized along deposited road boundaries, suggesting that the structurally weak regions of the printed architecture became more susceptible to opening during sliding. This observation is consistent with the comparable volume loss recorded after water conditioning.

The water-absorbed PLA-Wood surface in Figure 10D similarly showed abrasive grooves, elongated inter-road openings, and localized pull-out cavities. The persistence of particle- or void-associated damage explains why the volume loss remained close to that of untreated PLA-Wood.

Heat-treated PLA displayed the least severe worn morphology (Figure 10E). The deposited roads largely retained their continuity, while damage was restricted to fine abrasive grooves, small inter-road cavities, and limited debris accumulation. The absence of extensive flaking or large pull-out regions is consistent with the lowest volume loss observed among the PLA groups. Based on the plotted mean values, heat treatment reduced the PLA volume loss by approximately 20% relative to the untreated condition.

Thermal annealing can increase the crystallinity of FFF-manufactured PLA and modify its porosity, inter-road cohesion, and mechanical response. Accordingly, the better-preserved surface integrity observed here is consistent with increased resistance to plastic deformation and material detachment.

The heat-treated PLA-Wood given in Figure 10F retained clear abrasive grooves but exhibited a locally lifted surface layer and debris trapped beneath the delaminated region. Thus, heat treatment did not eliminate the heterogeneous damage associated with the composite structure. Nevertheless, compared with untreated PLA-Wood, large pull-out cavities and coarse, fragmented regions were less prevalent, consistent with the approximate 10% reduction in volume loss following heat treatment. The dominant wear mechanism may therefore be described as abrasive wear accompanied by localized subsurface or inter-road delamination.

The surface of heat-treated and subsequently water-absorbed PLA is shown in Figure 10G. In addition to abrasive grooves, a distinct crack-like discontinuity and local flake detachment were observed. These features indicate that abrasion was accompanied by surface-fatigue cracking and localized delamination. The reappearance of more pronounced cracking and flaking is consistent with the increase in volume loss from the HT to the HT + WA condition. Water conditioning, therefore, appears to have substantially reduced the wear-resistance improvement achieved by heat treatment in neat PLA.

In contrast, the heat-treated and subsequently water-absorbed PLA-Wood surface presented in Figure 10H showed localized pull-out cavities, matrix smearing, and directional abrasive grooves, but no extensive delaminated layer comparable to that observed in Figure 10F. Its volume loss remained close to that of the heat-treated PLA-Wood group and below those of the untreated and water-absorbed PLA-Wood groups. This indicates that the beneficial effect of heat treatment was at least partly retained after water absorbing in the PLA-Wood material. The principal wear mechanism was therefore abrasive wear combined with limited particle-rich-region pull-out, matrix deformation, and localized interfacial opening.

Overall, PLA-Wood exhibited a greater volume loss than neat PLA across all conditioning states. The SEM observations associate this behavior with the more heterogeneous worn morphology of PLA-Wood, including larger cavities, particle or interface-related pull-out, delamination, and debris accumulation. Heat treatment improved the wear resistance of both materials, although its effect was more pronounced in neat PLA. Subsequent water absorption largely eliminated this improvement in PLA but caused only a limited increase in the heat-treated PLA-Wood’s volume loss. These results indicate that the influence of the conditioning history on wear behavior depends on the material composition.

## 4. Conclusions

The tribological performance of FFF-printed polymer components should be evaluated by considering manufacturing parameters, post-processing, and service conditions as an integrated system rather than isolated factors. To address this issue, systematic sequential methodology was developed, beginning with the identification of the best-performing infill architecture among those investigated, followed by the identification of the best-performing heat-treatment conditions among those investigated, and concluding with the evaluation of water exposure effects on the tribological performance of PLA and PLA-Wood components. The findings indicate that the infill architecture significantly affects the tribological performance of both PLA and PLA-Wood specimens. Of all printing configurations examined, the rectilinear pattern with an 80% infill density yielded the lowest volumetric wear loss, measured at 17.97 ± 0.92 mm^3^ for PLA and 24.92 ± 0.63 mm^3^ for PLA-Wood. Heat treatment further enhanced wear resistance, decreasing volumetric wear loss by approximately 20% in PLA and 10% in PLA-Wood under the selected heat-treatment conditions (90 for 15 min for PLA and 90 for 45 min for PLA-Wood). Water absorption, however, substantially reduced the beneficial influence of heat treatment, particularly in PLA, whereas the improvement was largely retained in PLA-Wood. Scanning electron microscopy (SEM) analysis confirmed that abrasive wear was predominant under all conditions, and the observed variations in wear performance were closely due to changes in surface integrity, inter-road opening, localized pull-out, and delamination.

This study establishes a causal relationship among the processing history, environmental conditioning, wear performance, and the evolution of surface damage, thereby contributing to a comprehensive understanding of the tribological response in FFF-printed polymers. The proposed methodology is applicable to a broad range of engineering polymers and polymer composites, offering a practical approach to developing additively manufactured components with enhanced durability for wear-related applications.

## Figures and Tables

**Figure 1:**
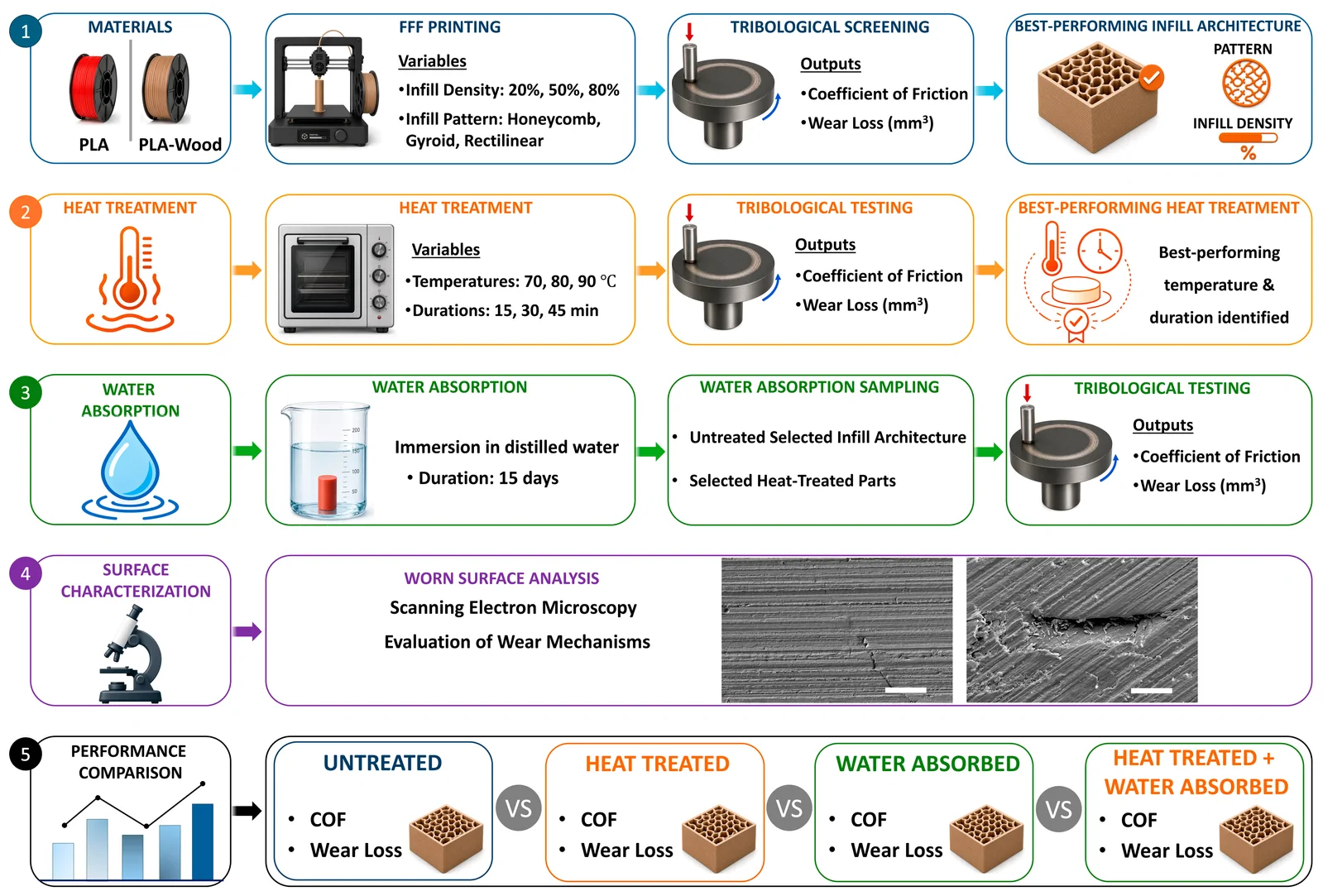
Experimental workflow showing the sequential identification of the best-performing infill architecture and heat-treatment conditions based on the tribological response of the specimens, followed by water absorption, worn surface characterization, and a comparison of the tribological behavior of all specimen groups.

**Figure 2:**
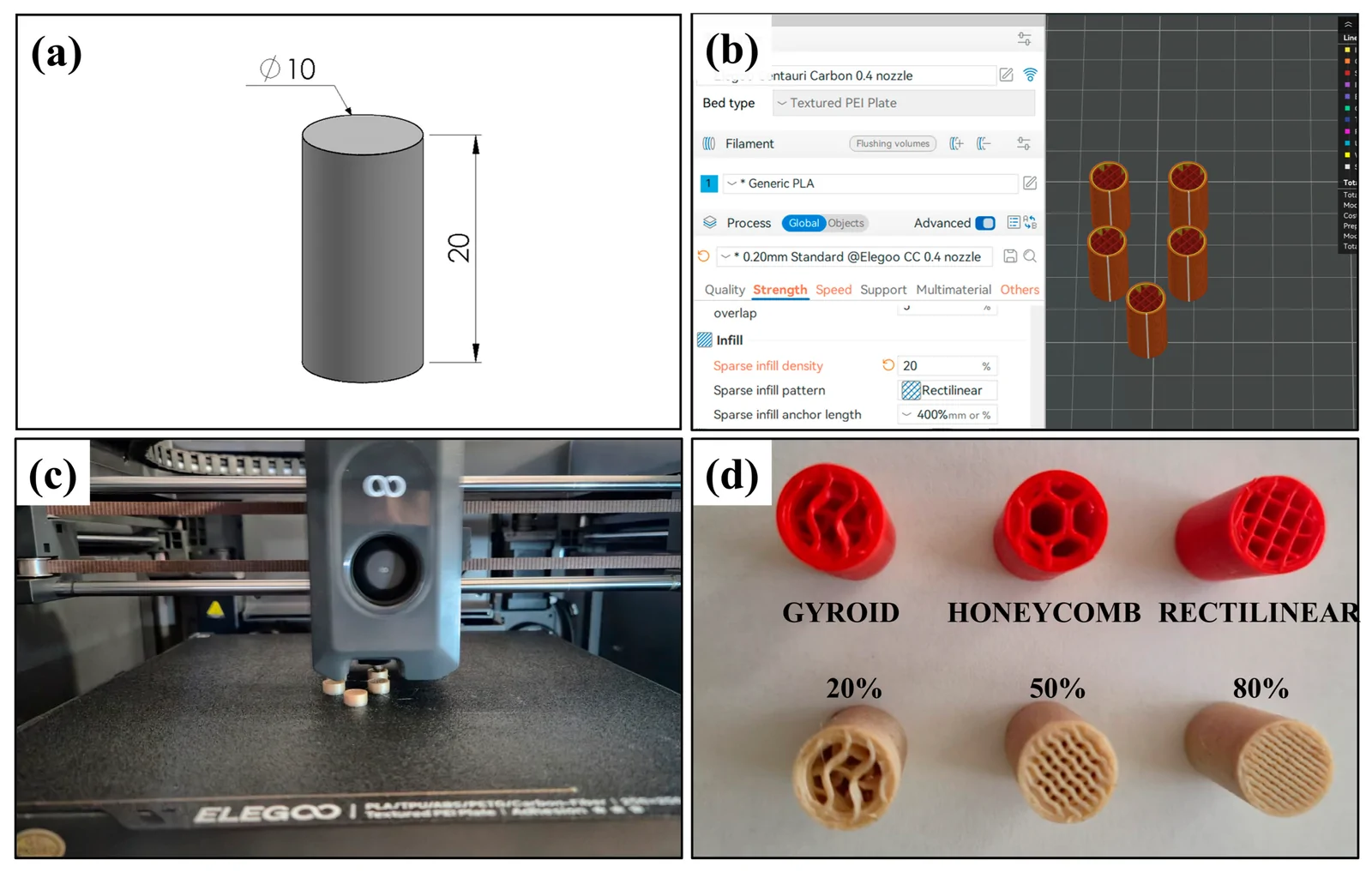
Preparation of wear test specimens. (**a**) Computer-aided design model of the ASTM G99 wear specimen designed in SolidWorks 2022, (**b**) slicing process, (**c**) FFF fabrication of the specimens, and (**d**) representative PLA and PLA-Wood wear test specimens.

**Figure 3:**
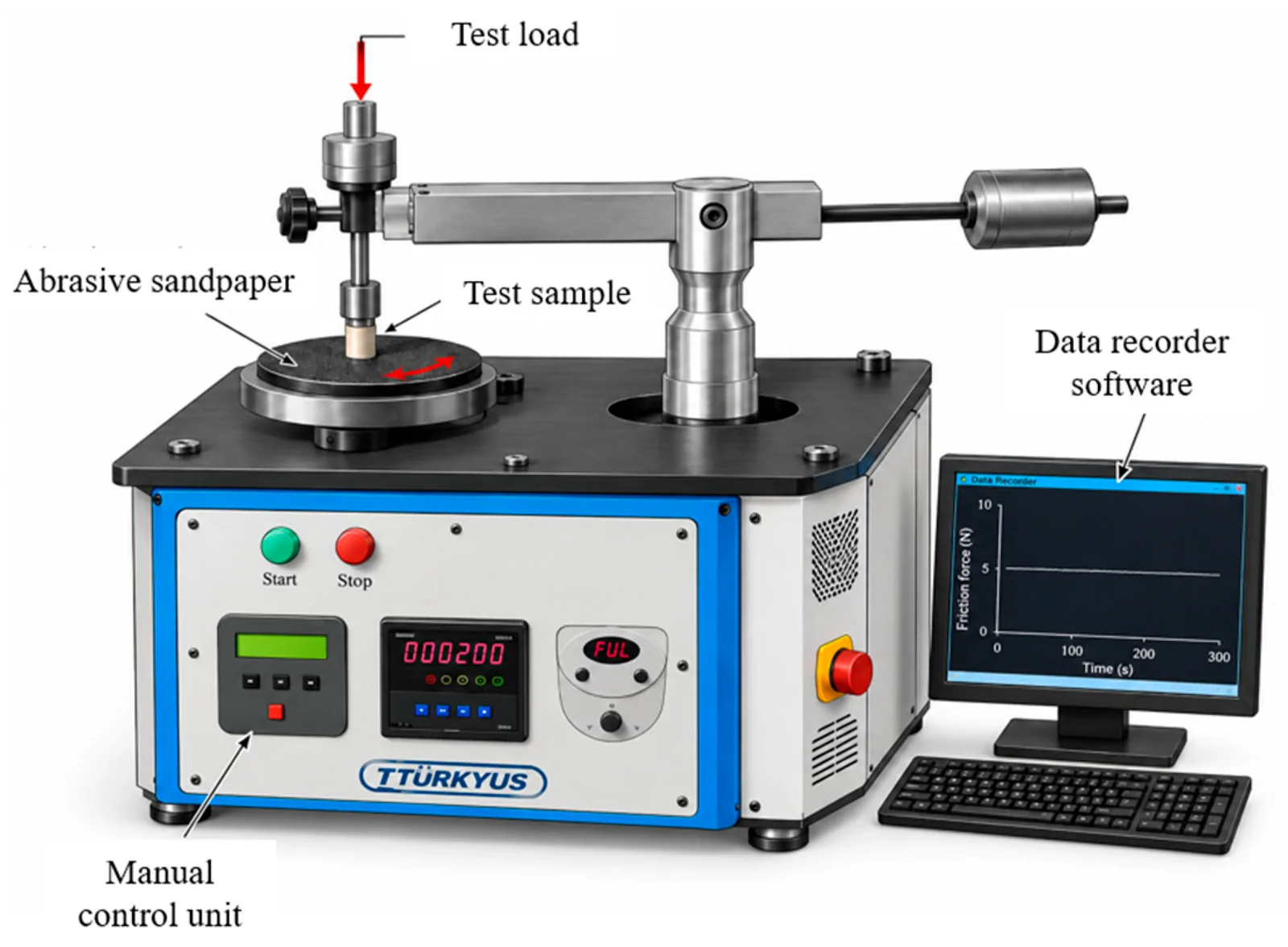
A schematic view of the wear test setup.

**Figure 4:**
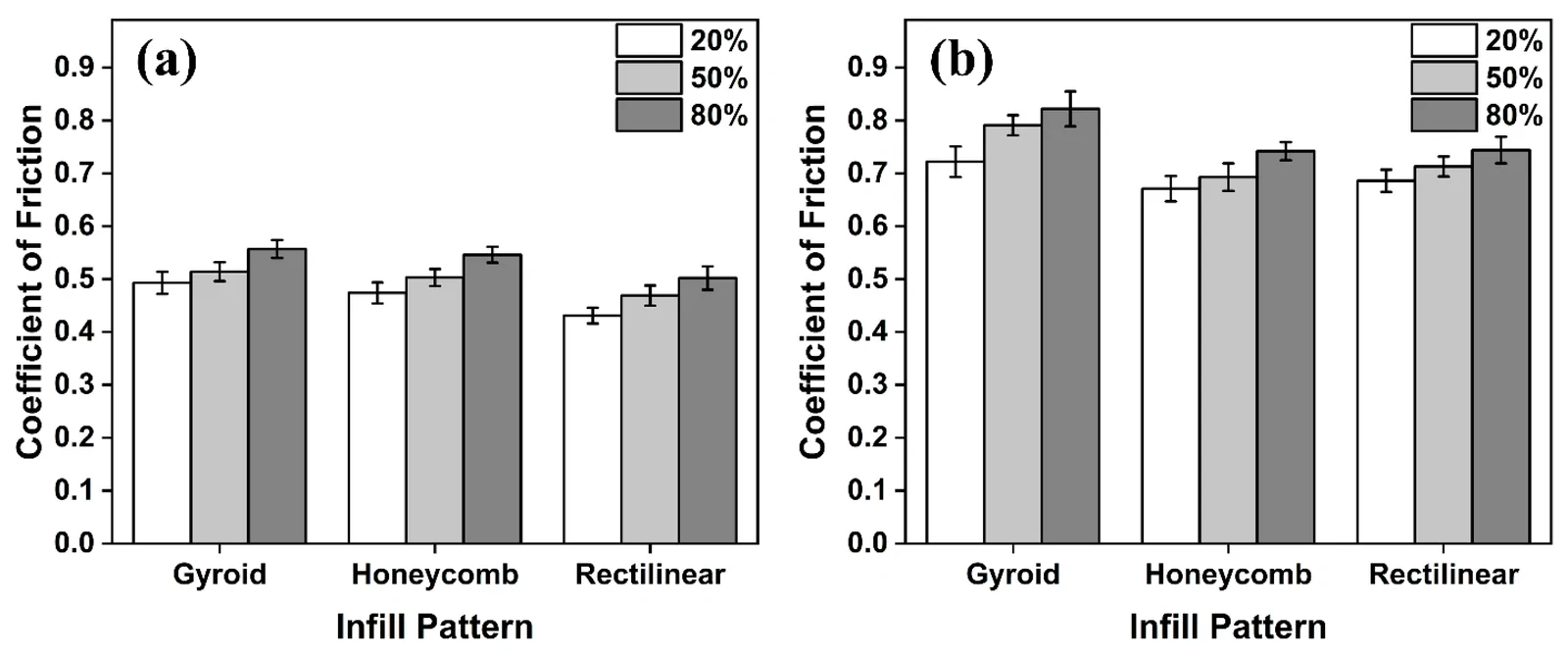
Coefficient of friction values of FFF-printed specimens with different infill densities and patterns: (**a**) PLA and (**b**) PLA-Wood.

**Figure 5:**
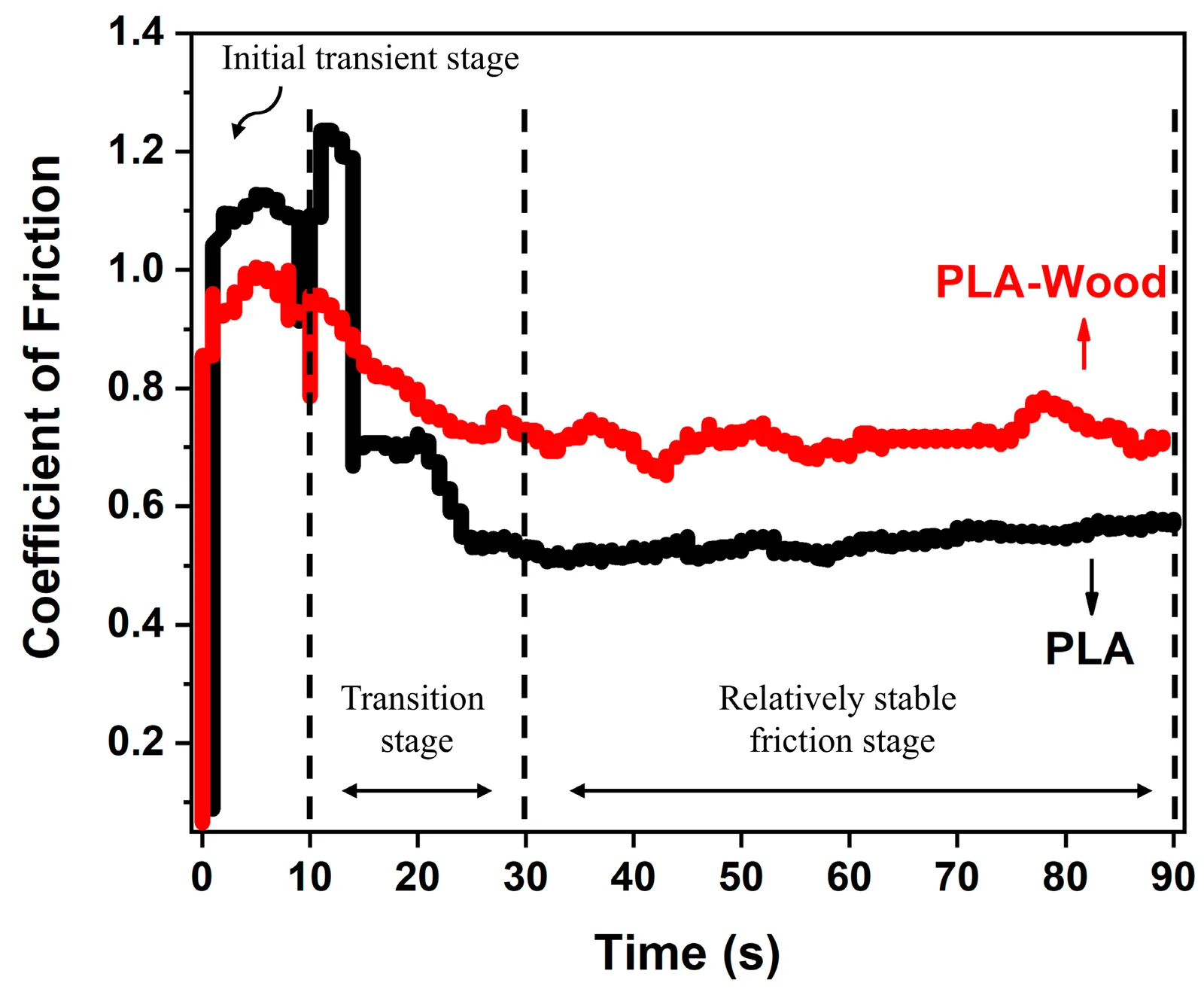
Representative COF–time curves of PLA and PLA-Wood specimens fabricated with 80% infill density and a rectilinear pattern.

**Figure 6:**
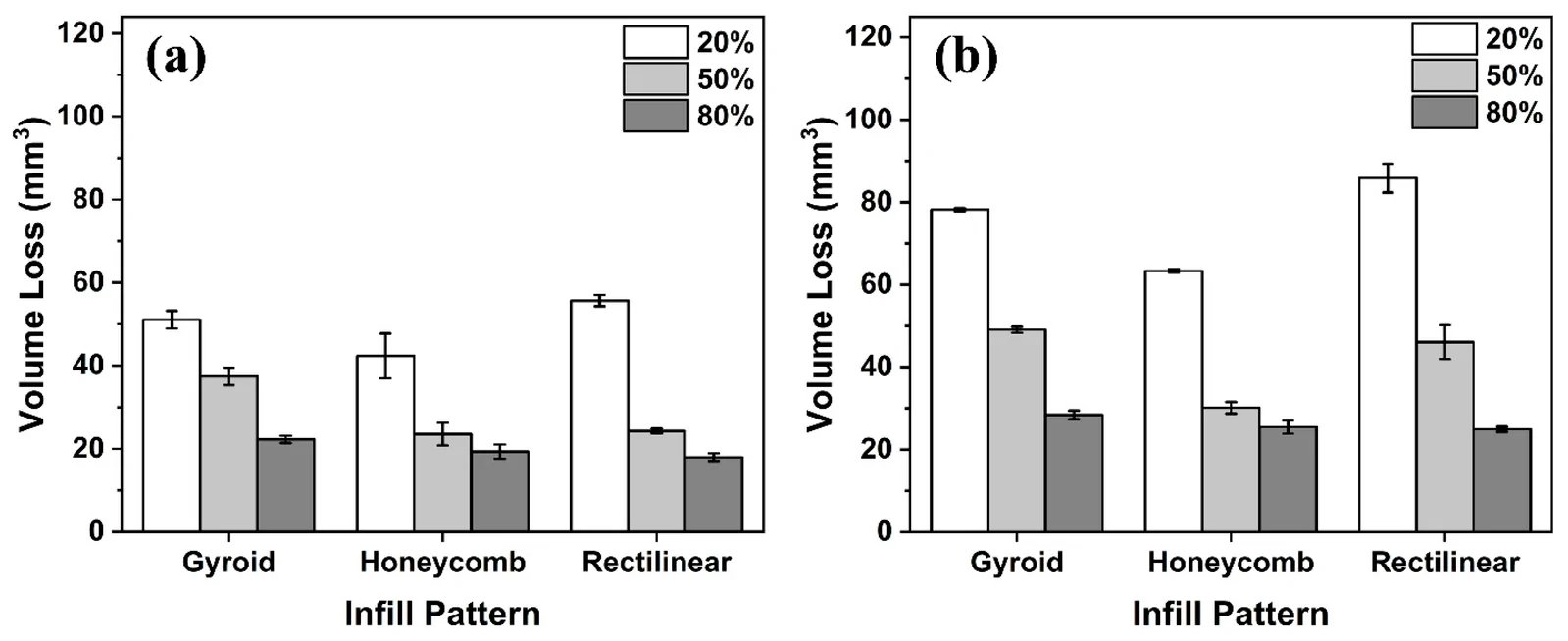
Volumetric wear loss of FFF-printed specimens with different infill densities and patterns: (**a**) PLA and (**b**) PLA-Wood.

**Figure 7:**
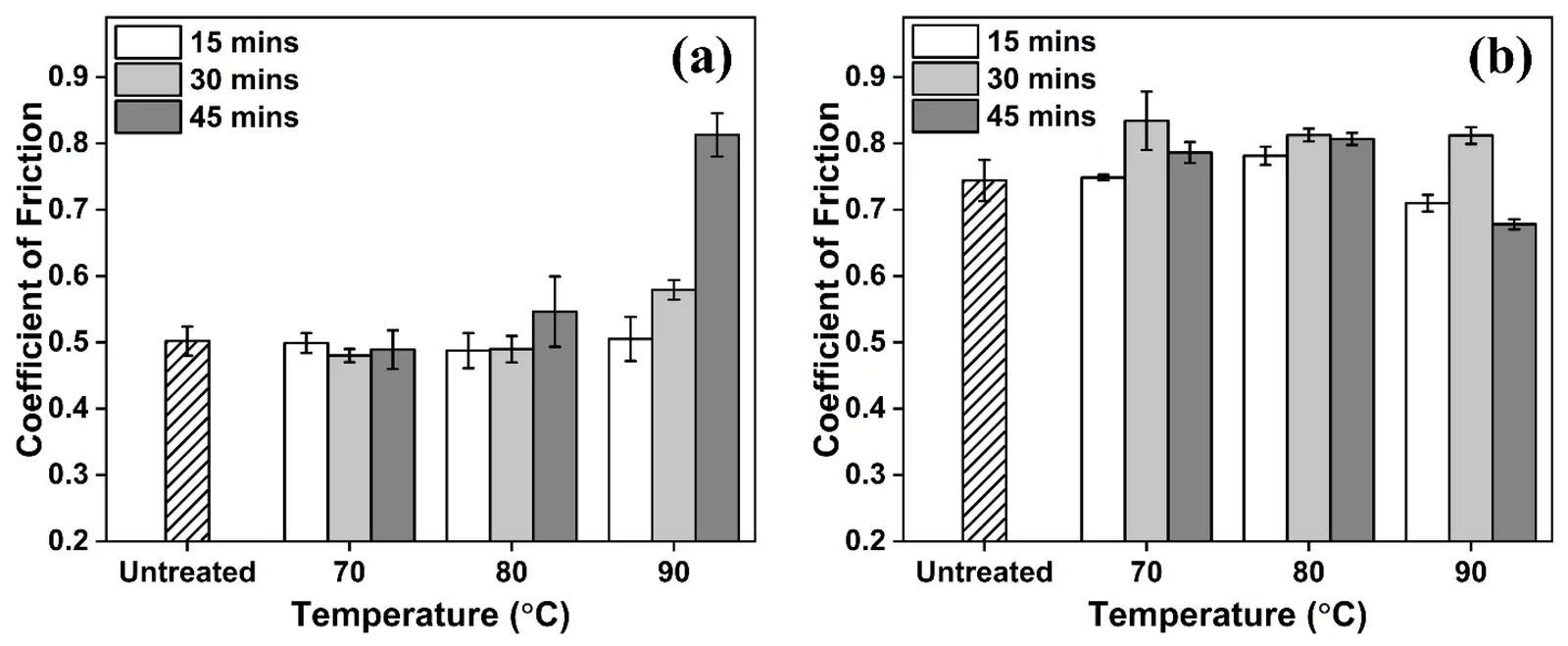
Effect of heat treatment on COF of FFF-printed specimens fabricated with the selected infill architecture (80% infill density and rectilinear pattern): (**a**) PLA and (**b**) PLA-Wood.

**Figure 8:**
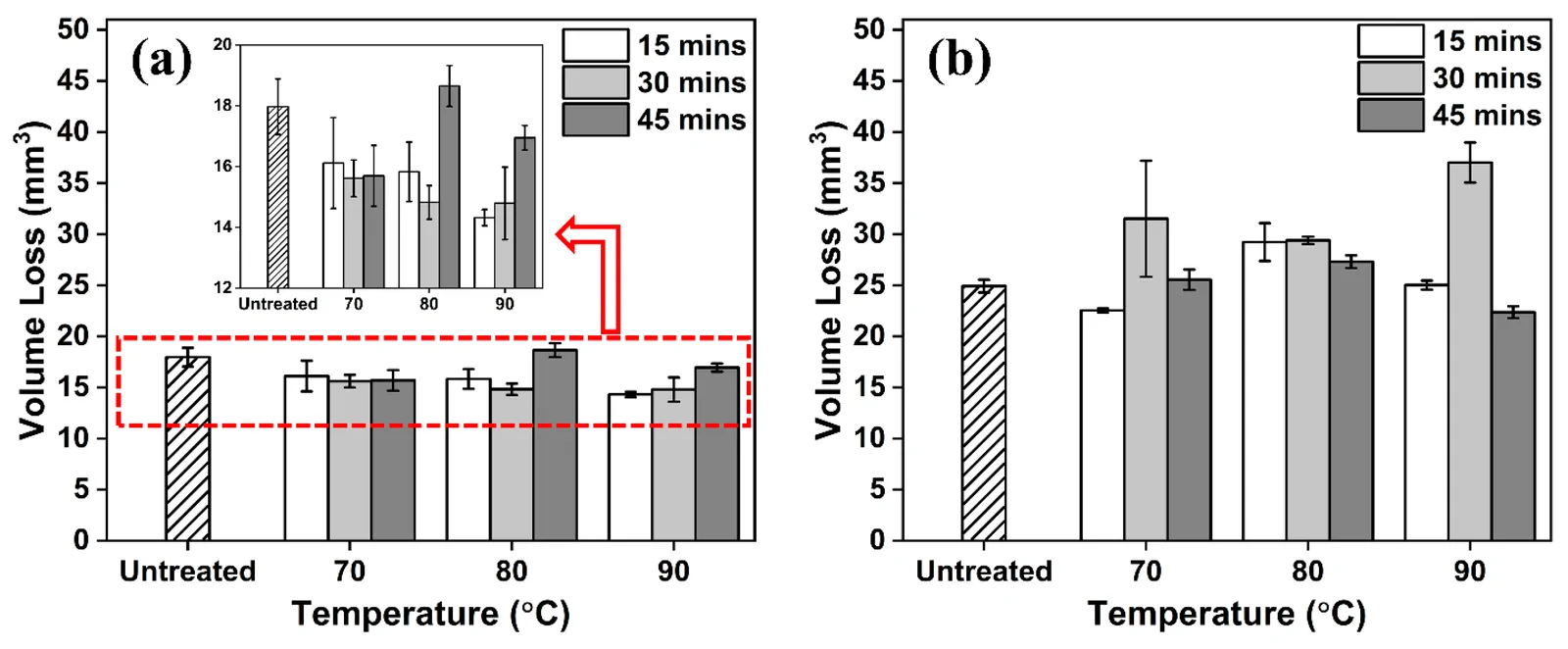
Effect of heat treatment on the volumetric wear loss of FFF-printed specimens fabricated with the selected infill architecture (80% infill density and rectilinear pattern): (**a**) PLA and (**b**) PLA-Wood.

**Figure 9:**
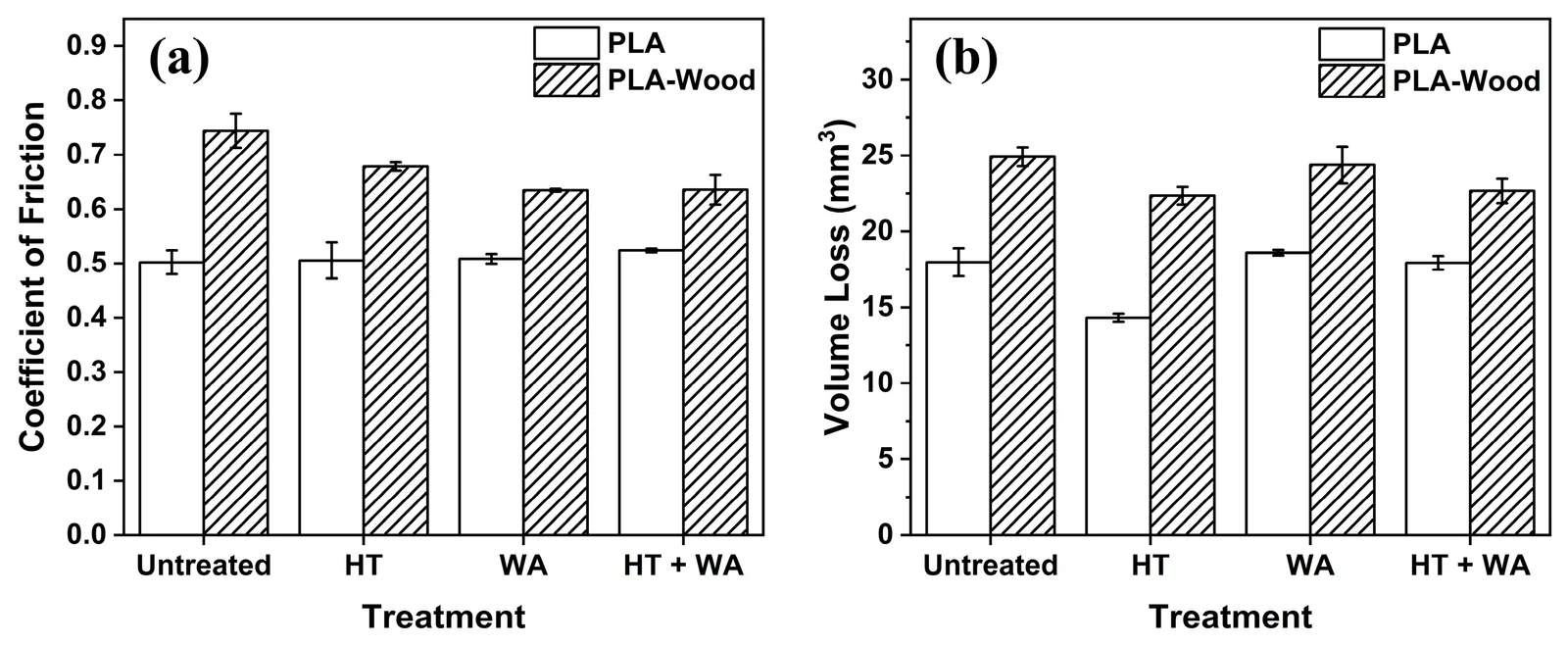
Comparison of tribological performance of untreated, heat-treated (HT), water-absorbed (WA), and heat-treated + water-absorbed (HT + WA) PLA and PLA-Wood specimens: (**a**) coefficient of friction and (**b**) volumetric wear loss.

**Figure 10:**
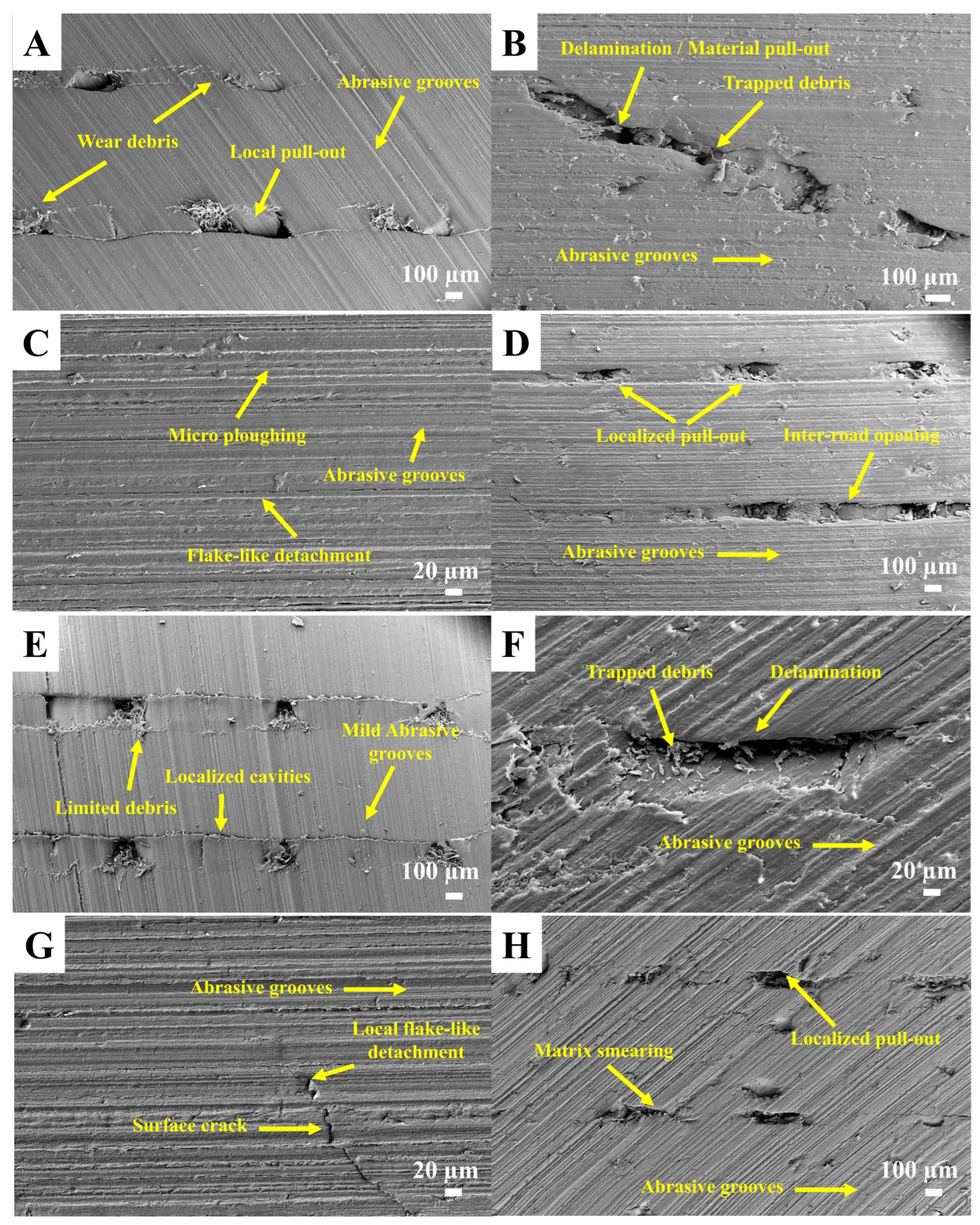
Worn surface analysis of FFF-printed PLA and PLA-Wood samples. (**A**) Untreated PLA, (**B**) untreated PLA-Wood, (**C**) water-absorbed PLA, (**D**) water-absorbed PLA-Wood, (**E**) heat-treated PLA, (**F**) heat-treated PLA-Wood, (**G**) heat-treated + water-absorbed PLA, (**H**) and heat-treated + water-absorbed PLA-Wood.

**Table 1:** Physical and mechanical properties, as reported by the manufacturer, and recommended processing conditions for PLA and PLA-Wood filaments utilized in this study.

Material Property (Unit)	Materials	
	PLA	PLA-Wood
Density (g/cm^3^)	1.23	n/a
Melt Flow Index (g/10 min)	17.3	12.2
Glass Transition Temperature (°C)	55–60	60–65
Tensile Strength (MPa)	56	45
Elastic Modulus (MPa)	2850	3000
Elongation at break (%)	7	4
Izod Impact Strength (kJ/m^2^)	14.2	4.2
**Recommended Processing Parameters**		
Extruder Temperature (°C)	200–230	210–240
Bed Temperature (°C)	0–60	0–60
Drying Conditions (°C/h)	30–40/2–4	30–40/2–4
Part Cooling Fan/Build Chamber	100%/No enclosure required	100%/No enclosure required

**Table 2:** Process parameters for 3D printing of PLA and PLA-Wood specimens used in this study.

Parameter	Value
Nozzle Diameter (mm)	0.4
Extruder Temperature (°C)	220
Bed Temperature (°C)	60
Layer Height (mm)	0.2
Printing Speed (mm/s)	50
Number of Contours	2
Build Orientation	Vertical
Raster Angle (°)	+45/−45
Support Structure	None
Adhesion Type	None
Fan (%)	100
Infill Density (%)	20/50/80
Infill Pattern	Gyroid/Honeycomb/Rectilinear

## Data Availability

The datasets generated and/or analyzed during the current study are available from the corresponding author upon reasonable request.

## Abbreviations

The following abbreviations are used in this manuscript:

FFF	Fused filament fabrication
AM	Additive manufacturing
PLA	Polylactic acid
PLA-Wood	Wood-filled polylactic acid
PETG	Polyethylene terephthalate glycol
PET	Polyethylene terephthalate
ABS	Acrylonitrile butadiene styrene
ASA	Acrylonitrile styrene acrylate
PA	Polyamide
PC	Polycarbonate
COF	Coefficient of friction
SiC	Silicon carbide
SD	Standard deviation
HT	Heat-treated
WA	Water-absorbed
